# Glycerophosphocholine and Glycerophosphoethanolamine Are *Not* the Main Sources of the *In Vivo* ^31^P MRS Phosphodiester Signals from Healthy Fibroglandular Breast Tissue at 7 T

**DOI:** 10.3389/fonc.2016.00029

**Published:** 2016-02-15

**Authors:** Wybe J. M. van der Kemp, Bertine L. Stehouwer, Jurgen H. Runge, Jannie P. Wijnen, Aart J. Nederveen, Peter R. Luijten, Dennis W. J. Klomp

**Affiliations:** ^1^Radiology, University Medical Center Utrecht, Utrecht, Netherlands; ^2^Radiology, Academic Medical Center, Amsterdam, Netherlands

**Keywords:** MRSI, ^31^P, relaxation time, 7 T, phosphodiester, breast, phospholipids

## Abstract

**Purpose:**

The identification of the phosphodiester (PDE) ^31^P MR signals in the healthy human breast at ultra-high field.

**Methods:**

*In vivo* ^31^P MRS measurements at 7 T of the PDE signals in the breast were performed investigating the chemical shifts, the transverse- and the longitudinal relaxation times. Chemical shifts and transverse relaxation times were compared with non-ambiguous PDE signals from the liver.

**Results:**

The chemical shifts of the PDE signals are shifted −0.5 ppm with respect to glycerophosphocholine (GPC) and glycerophosphoethanolamine (GPE), and the transverse and longitudinal relaxation times for these signals are a factor 3 to 4 shorter than expected for aqueous GPC and GPE.

**Conclusion:**

The available experimental evidence suggests that GPC and GPE are not the main source of the PDE signals measured in fibroglandular breast tissue at 7 T. These signals may predominantly originate from mobile phospholipids.

## Introduction

**Figure 1:**
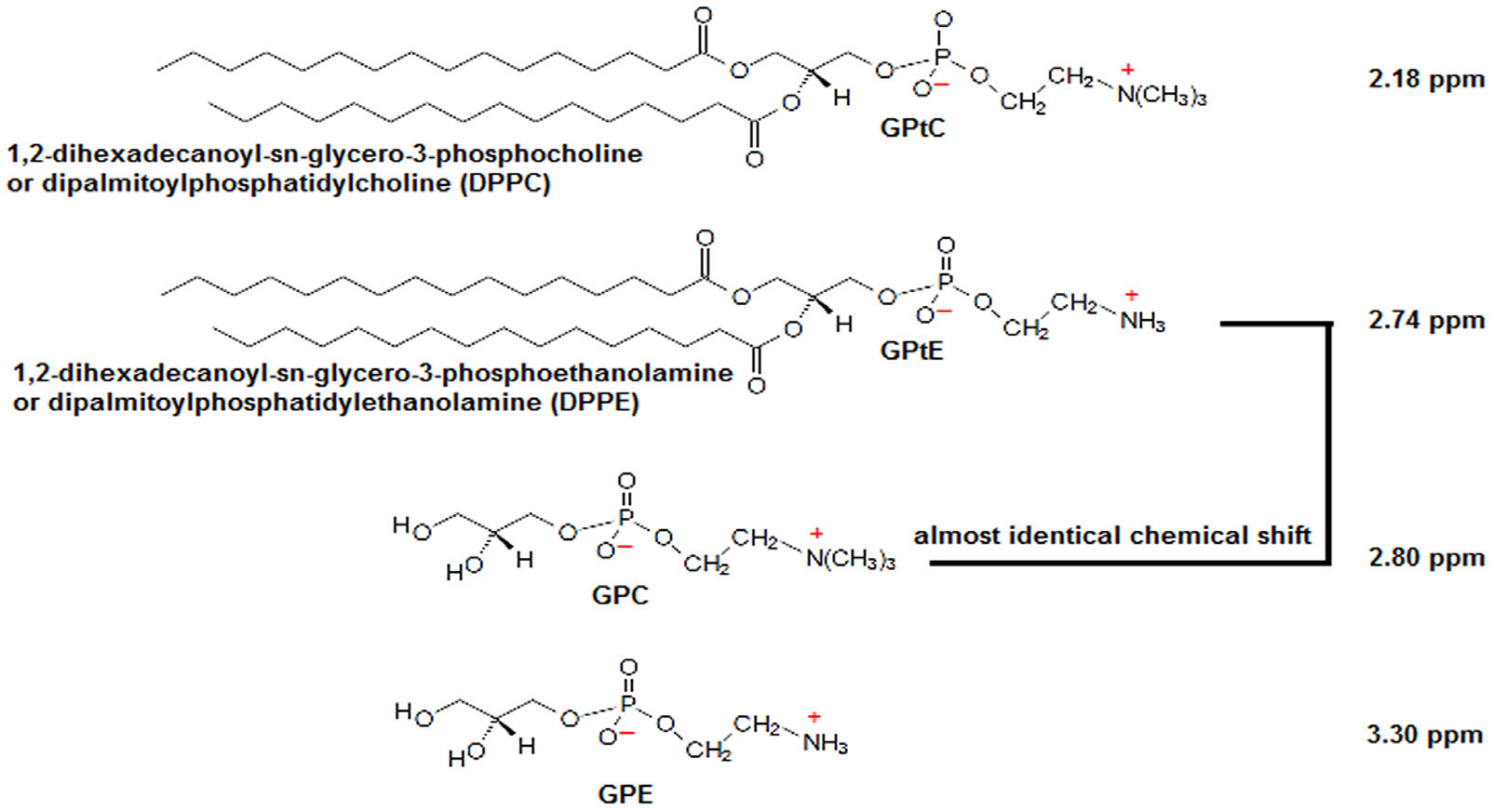
**Molecular structures of GPC, GPE, and their membrane phospholipids GPtC and GPtE**. Chemical shift of GPC is taken as a reference at 2.80 ppm. Chemical shift differences Δδ(GPtE−GPtC) = 0.56 ppm and (GPC−GPtC) = 0.62 ppm were calculated from high-resolution spectra (spectral resolution <0.02 ppm) by Schiller and Arnold (15), and Δδ(GPE−GPC) = 0.50 ppm from Payne et al. (16).

The phosphomonoesters (PME), phosphocholine (PC) and phosphoethanolamine (PE), and the phosphodiesters (PDEs), glycerophosphocholine (GPC) and glycerophosphoethanolamine (GPE), are involved in cell membrane metabolism. From *ex vivo* studies, it is known that the PC/GPC ratio goes up on malignant transformation of cells (1, 2), while the decrease of the PC/GPC ratio was shown to be a marker in predicting cancer treatment response in *ex vivo* NMR studies (2–4). *In vivo*, the total choline signal, which can be obtained by localized ^1^H MRS, has been shown to be a biomarker for malignancy and treatment response (5). In contrast to *in vivo* ^1^H MRS, where only a total choline signal can be observed, one can easily distinguish PME from PDEs with *in vivo* ^31^P MRS and even PE from PC and GPE from GPC, with ultra-high field ^31^P MRS (6). Besides higher spectral resolution, ultra-high field MRS comes with a higher signal-to-noise ratio that can be traded off for improving spatial resolution or to shorten scan time. High *in vivo* PME/PDE ratios, as measured with ^31^P MRS, have been shown to be indicative of cancer, while treatment response is often accompanied by a reduction in PME/PDE (7–12). However, in contrast to some *ex vivo* methods, where extraction techniques are used to separate aqueous pools of metabolites from lipid pools, *in vivo* methods will also obtain signals from membrane phospholipids (MPL) (13, 14). Moreover, as these MPL have chemical shifts similar to GPC, e.g., glycerophosphatidylethanolamine (GPtE) has almost identical chemical shift as GPC (15, 16) (the molecular structures and chemical shifts are shown in Figure 1) – *in vivo* distinction of these compounds is hampered.

At lower field strength (<2.5 T), *in vivo* ^31^P spectra of various tissues, e.g., breast (17, 18), brain (19–24), liver (25–28), and kidney (29, 30), show a large signal in the PDE chemical shift range, with its top between 2 and 3 ppm with respect to phosphocreatine (PCr) at 0 ppm. The full width at half max of this signal is dependent on the field strength, and the delay between excitation and acquisition, as, for instance, caused by phase encoding. It has been suggested (21, 25) that this membrane peak disappears almost completely at high-field strength due to enhanced relaxation by chemical shift anisotropy, leaving only the signals of the aqueous-soluble metabolites GPC and GPE. Nowadays, with high magnetic field human MRI systems becoming available, the origin of the PDE ^31^P MRS signal – whether GPC and GPE, and/or GPtC and GPtE – is of renewed interest.

Here, we show that the PDE signals measured *in vivo* in fibroglandular tissue of the human breast (^31^P signal from breast fatty tissue is below the detection limit) at 7 T are possibly signals from MPL, although their line widths suggest aqueous small molecules, such as GPC and GPE. Measurements are performed at 7 T to distinguish GPE from GPC and GPtE from GPtC. Adiabatic multi-echo spectroscopic imaging (AMESING) (31) and progressive saturation are used to identify the mobility of the molecules as reflected in the *T*_2_- and *T*_1_-values, respectively, to enable a distinction between the aqueous GPE and GPC from the more restricted MPL (GPtE and GPtC). Data are obtained in breast glandular tissue and compared to GPC and GPE metabolite signals as measured in liver tissue, all in healthy human volunteers *in vivo*.

## Materials and Methods

^31^P MRS measurements of glandular breast tissue were obtained from healthy volunteers using a dedicated breast coil (MR Coils BV, Drunen, The Netherlands) interfaced to a 7-T MRI system (Philips, Cleveland, OH, USA). Pulse-acquire and multi-echo acquisitions [AMESING (31)] were obtained with adiabatic RF pulses. Excitation was done with an adiabatic half passage (AHP) of 2 ms. For refocusing 4 ms *B*_1_ insensitive rotation pulses (BIR-4 180°) were used. The AHP excitation pulse had a frequency sweep of 10.0 kHz and the BIR-4 refocusing pulses a frequency sweep of 20.0 kHz. Both pulses had tangent frequency modulation and hyperbolic tangent amplitude modulation as described in Garwood and Ke (32). Pulses were driven with *γB*_1max_ = 1700 Hz. Transmitter offset on the ^31^P channel was set to 600 Hz with respect to the resonance of PCr. All chemical shifts reported here are referenced to PCr as standard at a chemical shift of 0.0 ppm, which is −2.48 ppm compared to 85% phosphoric acid. Frequency calibration of the scanner is done based on the water signal (the MR system uses a fixed ratio between ^31^P and ^1^H carrier frequency such that the proton signal for water corresponds to the ^31^P PCr signal). Measurements with the AMESING sequence were performed with a *T*_R_ of 6 s, 8 × 8 × 8 spherical acquired MRSI, 2 cm × 4 cm × 4 cm voxel sizes for the breast on five volunteers.

Both FID and symmetric echoes were acquired with 256 data points, and the spectral bandwidth for the acquisition of the FID was 17.0 kHz and for the echoes 8.5 kHz (echo spacing 45 ms) to maintain equal acquisition durations for FID and each half echo. Acquired data were spatially Hamming filtered and zero filled in the time domain to 8192 data points. To obtain high SNR spectra of the breast, the datasets of five volunteers (age range 24–30 years) were pooled and Pi-weighted based on the FID signal. Phosphorus metabolite *T*_1_-values in the breast were measured for five volunteers by means of progressive saturation with an adiabatic AHP pulse-acquire 1D MRSI sequence with *T*_R_ values in the range of 0.5–8 s, where the scan time was kept identical for each *T*_R_. The FID data were acquired with 512 data points and a spectral bandwidth of 8.2 kHz. A 1D MRSI encoded in the anterior–posterior direction was chosen to effectively suppress signals from the underlying pectoral muscles. Data were spatially Hamming filtered and subsequently zero filled in the time domain to 8192 data points. To obtain high SNR datasets for *T*_1_-fitting, the volunteers were measured two or three times and the data per volunteer were averaged. Before averaging, all spectra were aligned for Pi. Averaged spectra were spectrally fitted in JMRUI (33) using the AMARES algorithm (34), chemical shifts for the GPtE + GPC and GPtC resonances were fixed with a soft constraint to 2.77 ± 0.1 ppm and 2.18 ± 0.1 ppm and free but equal line width.

^31^P MRS liver measurements were also done with the AMESING sequence with 32 × 10 2D MRSI (feet–head direction unlocalized) voxel sizes 1 cm × 1 cm (echo spacing 40 ms) on five volunteers for the liver [data included from earlier study (35)], using a half volume coil (MR Coils BV, Drunen, The Netherlands). Other parameters and data post-processing and analysis were equal to those used in the breast measurements. Here, a 2D scheme with small AP and LR dimensions of the voxels was chosen to be able to exclude signal from muscle tissue, whereas the FH dimension is unlocalized but constrained by the coil sensitivity to encompass the liver but not beyond.

The study was approved by the local medical ethics review board (METC UMC Utrecht) and written informed consent was obtained from all volunteers.

## Results

**Figure 2:**
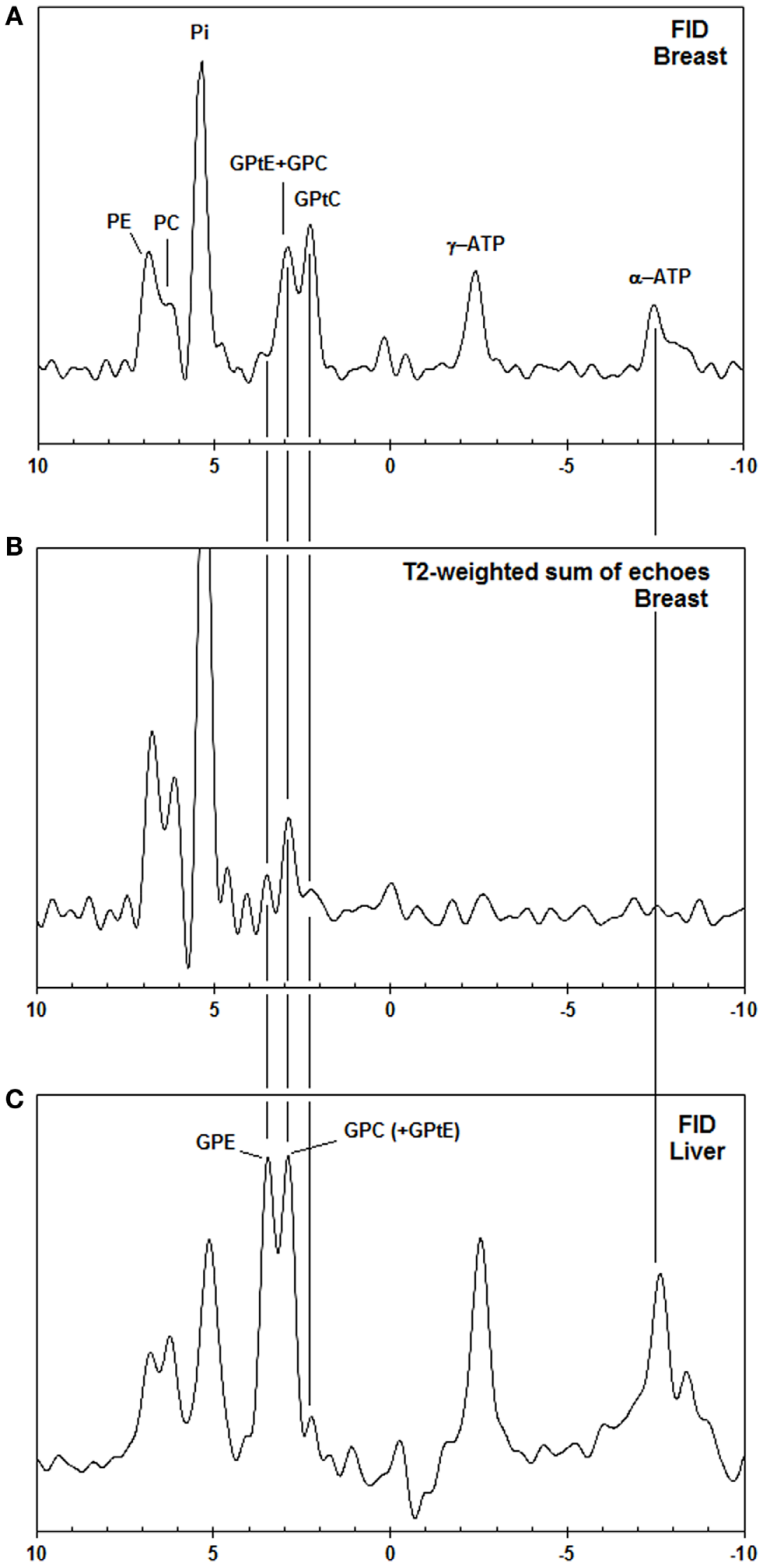
**(A)** Pulse acquire, **(B)** *T*_2_-weighted echo sum (*T*_2_ = 154 ms) ^31^P MR spectra [AMESING sequence (31)] from a voxel (2 cm × 4 cm × 4 cm) of the breast (average of five volunteers) scaled to the same noise, and **(C)** pulse-acquire ^31^P MR spectrum from the liver (average of five volunteers). Note that only the aqueous metabolites with long *T*_2_-values, such as PE, PC, Pi, and GPC get enhanced in the *T*_2_-weighted echo sum and that the chemical shifts of liver GPE and GPC do not match the PDE signals from the breast, but are shifted +0.5 ppm.

**Figure 3:**
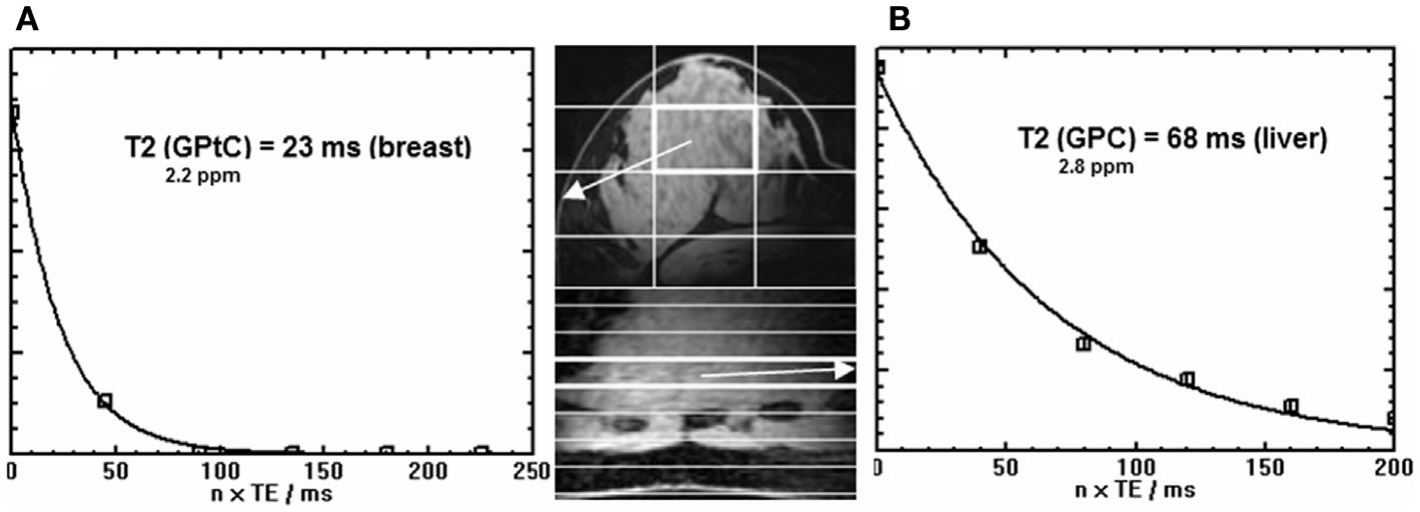
**Signal decay [as quantified by spectral fitting of FID and echoes with JMRUI (33)] as a function of time of the GPtC peak at 2.2 ppm obtained from (A) the breast (36) (echo spacing 45 ms) and (B) the true GPC peak from the liver (35) at 2.8 ppm (echo spacing 40 ms)**. Data were obtained by the AMESING sequence (31) and are averaged values for the group of volunteers. Note the threefold reduced *T*_2_ of the ^31^P spins of GPtC in the breast as compared to GPC in the liver. Images shown are (fast field echo) examples of breast and liver for one volunteer.

**Figure 4:**
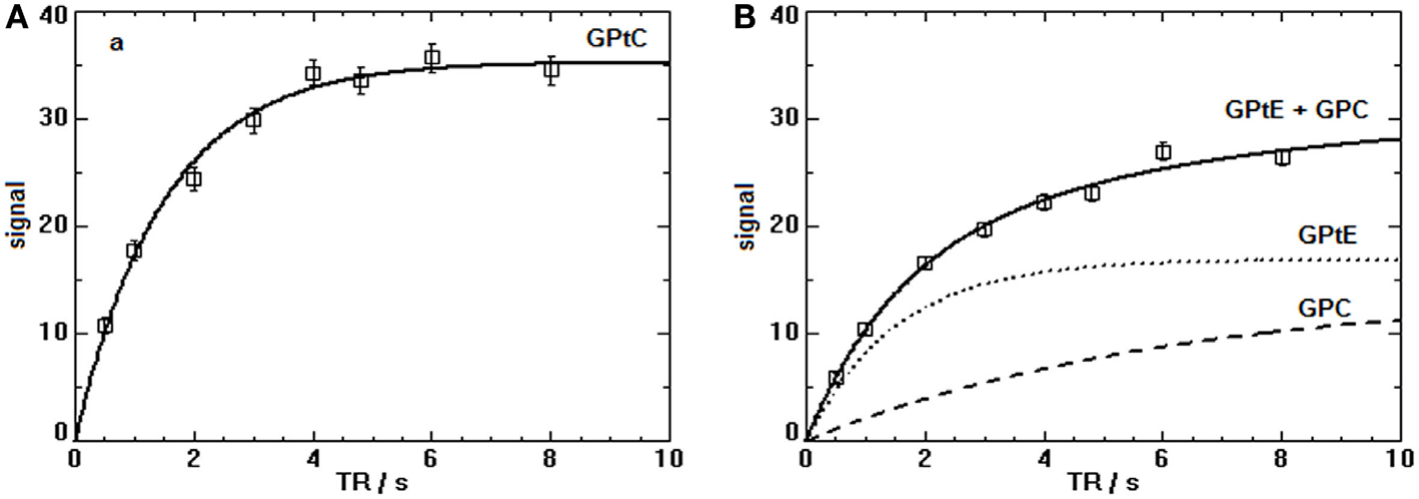
***T*_1_-fits of the progressive saturation measurements for the PDE signals (sum of five volunteers) in the breast at 7 T**. **(A)** The *T*_1_-value of the GPtC signal (mono-exponential decay) is 1.2 ± 0.3 s. **(B)** The (GPtE + GPC) signal for the five volunteers was fitted bi-exponentially with a fixed short *T*_1_ component for GPtE taken equal to GPtC leading to a *T*_1_ for GPC of 3.2 ± 0.6 s.

In Figure 2, the spectra of a voxel of the breast (average spectrum of five volunteers) and the liver (five volunteers) are shown. Due to limited bandwidth of the adiabatic pulses only the spectral range from +10 to −10 ppm is shown. The chemical shifts of the GPC and GPE signals in the liver (Figure 2C) do not correspond to the signals observed in the breast spectra (Figures 2A,B), which are usually labeled GPC and GPE, while the chemical shifts of the other metabolites (PE, PC, Pi, γ-ATP, α-ATP) in liver and breast do match. Figures 2A,B show, for a voxel of breast glandular tissue, the average FID and the average *T*_2_-weighted echo-sum spectra using a fixed *T*_2_ weighting of 154 ± 5 ms (36), scaled to the same noise. Note that the signal intensities of the peaks labeled PE, PC, Pi, and (GPtE + GPC) increase, or at least do not decrease, in the echo-sum spectrum as compared to the FID spectrum of the breast, while the signals of GPtC and ATP, with known short *T*_2_-values, do decrease in the echo-sum spectrum. Unlike ATP, the short apparent *T*_2_ for GPtC (and GPtE) is not the result of homonuclear coupling. In Figure 3, a comparison is made between *T*_2_ fits obtained for the PDE signal at 2.2 ppm (labeled GPtC) from the breast (a) and the 2.8 ppm signal from the liver (labeled GPC), showing almost a factor 3 lower *T*_2_ for the GPtC signal from the breast. Figure 4 shows the *T*_1_-fits for the average signals of (GPtE + GPC) and GPtC from fibroglandular breast tissue as measured in the five volunteers. The (GPtE + GPC) signal is fitted bi-exponentially with a short *T*_1_ component for GPtE (taken equal to GPtC, *T*_1_ = 1.2 s) The *T*_1_-value that is fitted for the long *T*_1_ component GPC is 3.2 s.

## Discussion

Phosphorous spectra from the breast and liver as shown in Figure 2 do not match to the chemical shift of the PDE signals. As the chemical shift of GPC and GPE are hardly pH sensitive, but Pi and to a lesser extent also PE, PC, and γ-ATP are (37), pH differences between liver and breast may influence chemical shift. If we would shift the breast spectrum by +0.5 ppm to match the PDE signals between breast and liver, this would correspond to a shift in pH of +0.4 units based on Pi chemical shift. This pH difference is unreasonably large, moreover expressing the change in chemical shift of +0.5 ppm for PE, PC, and γ-ATP in pH units is either not possible, or goes beyond any physiologic condition. Therefore, it seems likely that the metabolite signals in the breast that do not match those in the liver are (GPtE + GPC) and GPtC. Healthy liver is known to show high signals of GPC and GPE in ^31^P MRS *in vivo* and also in *ex vivo* perchloric acid extracts (38, 39). The peak labeled GPtC in the liver spectrum of Figure 2C is sometimes referenced to as (potentially) phosphoenolpyruvate (28, 40). However, it does not show up in ^31^P MRS perchloric acid liver extract studies (38, 39), even though phosphoenolpyruvate is sufficiently soluble in an aqueous phase.

The most likely reason for the nearly constant (GPtE + GPC) signal over FID and echo sum (Figures 2A,B) is that aqueous GPC, with a relatively long *T*_2_, increases in the echo sum, while GPtC, just like GPtE, decreases in intensity due to short *T*_2_.

A recent lipidomic profiling study on healthy mammary epithelial and breast cancer cells (41) has shown that in the membranes of healthy mammary epithelial cells the concentration of GPtC is approximately twice the concentration of GPtE. If we assume that the *T*_2_-weighted echo-sum signal at the chemical shift of GPtE + GPC in Figure 2B is indeed all GPC and we assume a similar *T*_2_ for GPC and PE and PC, then we can calculate the GPtC to GPtE ratio by subtraction of the GPC contribution in Figure 2A. This leads to a GPtC to GPtE ratio of 2, in close agreement with the lipidomic profiling study. Minor contributions from sphingomyelin and glycerophosphatidylserines, seen in the lipidomic profiling study will probably add to the *in vivo* ^31^P MR signal of GPtE and glycerophosphatidylinositol to the *in vivo* ^31^P MR signal of GPtC, not altering the ratio substantially. Chemical shift differences for these different phospholipids are reported by Schiller and Arnold (15).

The ^31^P *T*_2_ values in liver can be low due to the presence of iron, for instance, in the form of ferritin, which is known to increase the relaxivity of water (42). *In vivo* values for *T*_2_ of ^31^P metabolites (PE, PC, Pi, GPE, GPC) in the liver ranging between 37 and 71 ms have recently been measured at 7 T in our hospital in a group of five healthy volunteers (35). For the breast, however, we measured that the *T*_2_ of the ^31^P spins at the chemical shift of GPtC (36) is even a factor 3 shorter than the *T*_2_ of the ^31^P spins of GPC in the liver (35), as shown in Figure 3. In fact, compared to the reported *T*_2_ values – measured at 7 T – of PDEs in calf muscle [*T*_2_ = 314 ms (43), 375 ms (31)] or of PC and PE in the breast, the *T*_2_ value of GPtC we measured is almost an order of magnitude lower. Spectral fitting of the FID spectrum of Figure 2A, simplified by equal line widths for PE, PC, Pi, and equal linewidths for the PDE signals shows an additional linewidth for the PDE signals of 9 Hz, which is close to the calculated value of 12 Hz when considering the measured *T*_2_ of 23 ± 1 ms (35) and a *T*_2_ for the PMEs and Pi of ~ 160 ms.

The low signals for GPE and GPC in the echo-sum spectra of the breast are corroborated by an *in vitro* extract study on breast tumors by Smith et al. (14), where it was shown that GPE and GPC concentrations are low in non-necrotic breast tumors and that, at low field, PDE signals observed *in vivo* are mainly from phospholipids. A recent LC MS study by Mimmi et al. (44) showed a very low average concentration of only 0.04 mmol/kg GPC in three healthy fibroglandular breast tissue samples.

Another reason to suspect that the dominant PDE signal we observe in the breast at 7 T originates from mobile lipid structures is based on the results of the *T*_1_-measurements of the PDE signal in the breast, as depicted in Figure 4. Here, the *T*_1_ of GPtC was fitted mono-exponentially leading to 1.5 ± 0.1 s and the signal of GPtE + GPC was fitted bi-exponentially with the *T*_1_ of the GPtE component fixed equal to the *T*_1_ of GPtC. For the signal of the aqueous GPC, this leads to 6 ± 2 s. The fitted *T*_1_ of mobile GPtC is three- to fivefold lower than that reported for GPC and GPE in calf muscle and brain at 7 and 3 T (43, 45, 46). A value of 1.4 s for the *T*_1_ of the ^31^P MRS signal of dipalmitoylphosphatidylcholine (DPPC) vesicles with an average diameter of 100 nm has been measured by Klauda et al. (47) above the phase transition temperature. For multi-lamellar dispersions of dimyristoylphosphatidylcholine (DMPC), a value around 1 s has been measured just above the phase transition temperature by Dufourc et al. (48). The *T*_1_-value of 6 s fitted for the GPC component, Figure 4B, agrees well with the *T*_1_-values for GPC reported at 3 T and 7 T for calf muscle and brain ranging from 4 to 7.8 s (43, 45, 46).

All presented data throughout this paper is based on average spectra of the group of volunteers. This has the advantage that it maximizes signal to noise and enables the most reliable relaxation time fitting. A short coming is that individual physiological differences between volunteers are averaged out.

The fitted linewidth of the two overlapping PDE signals of the breast spectrum depicted in Figure 2A is 58 Hz (i.e., 0.5 ppm). Bulk phospholipid bilayers show broad asymmetrical lineshapes (several tens of ppm) caused by large chemical shift anisotropy (49). Therefore, if the very sharp PDE resonances that we observe are from MPL, then these MPL must be highly mobile phospholipids, for which chemical shift anisotropy and dipolar couplings are sufficiently averaged out. Especially at ultra-high field, relaxation by chemical shift anisotropy that goes with the square of the field causes additional line broadening as compared to spectra recorded at lower field strength. Highly mobile phospholipids can be found in small-sized vesicles (<50 nm) (50), and in large arrays of lipidic particles (51), inter-lamellar attachments (52), and inverted cubic structures (52–54) within the lipid bilayer.

A rough estimate of the percentage of *in vivo* visible mobile phospholipids at 7 T can be made as follows. The ratio of PE to PC is ~2 and the PME to PDE ratio is ~1 (Figure 2A). A weighted average of PC concentrations measured in healthy breast tissue by Mimmi et al. (44) is 0.08 mmol/kg, with PE/PC = 2 (Figure 2A), this leads to a PME concentration of ~ 0.2 mmol/kg. Most of the PDE signal is from mobile phospholipids (Figures 2A,B). The total concentration of phospholipids in human tissues is in the range of 17–83 mmol/kg (55). With a signal ratio of PDEs to PMEs in breast glandular tissue of 1.4 at *T*_R_ = 6 s (35) and a *T*_1_ of PMEs of 5 s (56) and a *T*_1_ of mobile phospholipids of 1.5 s, the total concentration of *in vivo* visible mobile phospholipids in the human breast at 7 T is also of the order of ~0.2 mmol/kg. This leads to a crude estimate of the visible mobile phospholipid fraction at 7 T of 0.2–1.2%.

## Conclusion

The PDE signals from the breast, as measured with MRSI techniques at 7 T *in vivo*, show aberrant behavior from aqueous GPE and GPC. The *T*_1_ and *T*_2_ relaxation values for these PDE signals are too short to represent true aqueous GPC and GPE. In addition, the chemical shifts of these PDE signals do not correspond to GPE and GPC, but are shifted −0.5 ppm with regard to these, and correspond to chemical shift values of GPtE and GPtC. These PDE signals could originate from mobile lipid structures such as small vesicles with diameters ≤50 nm, large arrays of ILAs or large domains of inverted cubic phases within the lipid bilayer. As the PC over GPC ratio is used as a biomarker in breast cancer research, the *in vivo* obtained value will be contaminated with signal from GPtE – having a similar chemical shift as GPC – or the GPtC peak may be erroneously assigned as GPC.

## Author Contributions

WK: study design, MR measurements, data analyses, first draft of typescript; BS: MR measurements, critical revision of typescript; JR: MR measurements, data analysis, critical revision of typescript; JW: study design, critical revision of typescript; AN: study, design, critical revision of typescript; PL: study design, critical revision of typescript; DK: study design, critical revision of typescript. All authors approved the final version of the manuscript.

## Conflict of Interest Statement

The authors declare that the research was conducted in the absence of any commercial or financial relationships that could be construed as a potential conflict of interest.

## Abbreviations

AMESING, Adiabatic Multi-Echo Spectroscopic ImagING; CSA, chemical shift anisotropy; DPPC, dipalmitoylphospatidylcholine (1,2-dipalmitoyl-sn-glycero-3-phosphocholine); DMPC, dimyristoylphospatidylcholine (1,2-dimyristoyl-sn-glycero-3-phosphocholine); GPC, glycerophosphocholine; GPE, glycerophosphoethanolamine; GPtC, (diacyl-)glycerophosphatidylcholine; GPtE, (diacyl-)glycerophosphatidylethanolamine; ILA, inter-lamellar attachment; MPL, membrane phospholipids; PC, phosphocholine; PE, phosphoethanolamine; Pi, inorganic phosphate; PCr, phosphocreatine.
